# Evaluating Spin Bias in the Abstracts of Systematic Reviews and Meta-Analyses on Robotic-Assisted Total Hip Arthroplasty: A Systematic Review

**DOI:** 10.1177/15563316261430336

**Published:** 2026-04-09

**Authors:** Ayomide Michael Ade-Conde, Hassaan Abdel Khalik, James Abesteh, Wayne E. Moschetti, Olufemi R. Ayeni

**Affiliations:** 1 School of Medicine, Royal College of Surgeons in Ireland, Dublin, Ireland; 2 Division of Orthopaedic Surgery, McMaster University, Hamilton, ON, Canada; 3 Department of Orthopaedics, Dartmouth Hitchcock Medical Center, Lebanon, NH, USA

**Keywords:** total hip arthroplasty, robotic-assisted, methodology, spin bias, quality assessment, systematic review

## Abstract

**Background:**

Interest in robotic-assisted total hip arthroplasty (RA-THA) continues to grow, despite inconsistent evidence on its effectiveness.

**Purpose:**

We sought to assess for the presence of spin bias in abstracts of systematic reviews and meta-analyses (SRMAs) comparing RA-THA to conventional total hip arthroplasty (C-THA).

**Methods:**

We conducted a systematic review of studies identified in a comprehensive search of MEDLINE, EMBASE, and the Cochrane Database of Systematic Reviews from each database’s inception to June 23, 2025. Search terms included “robotic,” “hip,” “arthroplasty,” “systematic review,” and “meta-analysis.” Inclusion criteria were (1) SRMAs evaluating outcomes of RA-THA, (2) studies comparing RA-THA to conventional techniques, and (3) publications in English. Studies were excluded if they (1) assessed RA-THA with other robotic-assisted joint arthroplasty procedure, or (2) assessed RA-THA surgery alongside other technologies. Abstracts were assessed for the 15 most severe forms of spin as defined by Yavchitz et al. Methodological quality was assessed using the AMSTAR 2 (A Measurement Tool to Assess Systematic Reviews-2) tool.

**Results:**

Twelve SRMAs were eligible for inclusion. Eleven SRMAs (91.7%) contained at least 1 of the 15 most severe forms of spin. Type 3 spin (*selective reporting of efficacy outcomes favoring the intervention*) was the most common (58%), followed by type 5 (*conclusion claims the beneficial effect of the experimental intervention despite high risk of bias in primary studies*; 50%), and type 11 (*conclusion focuses selectively on statistically significant efficacy outcome;* 33.3%). No differences in study characteristics were found between SRMAs with and without the aforementioned types of spin. All SRMAs were rated as “critically low” in methodological quality, with most not reporting funding source of included studies (91.7%).

**Conclusion:**

This systematic review found there is a high prevalence of spin bias in abstracts of SRMAs evaluating RA-THA. The most common type of spin involved selective reporting of outcomes favoring RA-THA (type 3). Future SRMAs can minimize spin by presenting balanced reporting of efficacy, or lack thereof, in their abstracts and strengthen methodological rigor by consistently reporting study funding sources.

**Level of Evidence:**

Level IV, Systematic review of level-I to level-IV studies.

## Plain Language Summary

Evaluating spin bias in the abstracts of systematic reviews and meta-analyses on robotic-assisted total hip arthroplasty

**Why was the study done?** There continues to be growing interest in the use of robotics in total hip arthroplasty, despite the evidence remaining contradictory. Systematic reviews and meta-analyses (SRMAs) allow researchers to compile and synthesize evidence on a topic of interest. Importantly, spin bias may occur in the abstracts of SRMAs. Spin bias is defined as presenting the evidence more favorably than what is actually demonstrated by the data.

**What did the researchers do?** The research team conducted a systematic review of the literature to identify existing SRMAs on robotic-assisted total hip arthroplasty (RA-THA). Reviewers assessed each study for the presence of spin bias based on the 15 most severe forms identified by Yavchitz et al. The methodological quality of eligible SRMAs was also evaluated using the AMSTAR-2 tool.

**What did the researchers find?** Twelve SRMAs were included in the assessment of spin bias and methodological quality. Eleven of the 12 SRMAs (91.7%) contained at least one of the 15 types of spin. Type 3 spin (selective reporting of efficacy outcomes favoring the intervention) was the most common (58%), followed by type 5 (claiming a beneficial effect of the intervention despite high risk of bias in primary studies; 50%) and type 11 (focusing selectively on statistically significant efficacy outcomes; 33.3%). Methodological quality assessment found that all SRMAs were rated as “critically low.”

**What do the findings mean?** Findings from this study indicate that nearly all SRMAs on RA-THA present their results more favorably than the underlying evidence supports. This was most commonly achieved by selectively reporting outcomes that favored RA-THA. Future SRMAs on this topic should strive for greater balance in their presentation, ensuring that both benefits and limitations are accurately represented.

## Introduction

Conventional total hip arthroplasty (C-THA) has long been the gold standard for treating end-stage hip osteoarthritis.^1^ Recently, robotic-assisted total hip arthroplasty (RA-THA) has garnered interest as an innovation promising improvements in implant positioning and patient-reported outcome measures (PROMs). Nonetheless, evidence remains conflicted on the efficacy of RA-THA compared that of C-THA.^2,3^ There is ongoing debate on whether RA-THA results in significant improvements in PROMs,^3^ clinically relevant optimization of implant positioning,^4^ and/or reductions in complications (eg, dislocations).^2^

Despite inconsistent evidence, public interest in robotic-assisted total joint arthroplasty (TJA) continues to grow.^5^ When faced with fragmented and conflicting evidence, systematic reviews and meta-analyses (SRMAs) may offer clinicians an up-to-date consolidation and critical appraisal of the evidence. Nevertheless, SRMAs are prone to their own biases, most notable of which is “spin” bias, in which results are framed more favorably than the evidence suggests. As described by Yavchitz et al,^6^ spin bias can be present in abstracts in various forms, broadly categorized as misleading reporting, misleading interpretation, and inappropriate extrapolation of results. For example, a common spin bias identified in orthopedic surgery SRMAs is the selective reporting or overemphasis of beneficial outcomes, which is known as “type 3 spin.”^7,8^ Spin bias can significantly influence readers’ interpretation of results, particularly in abstracts, which present the first impression of a review and may be the only accessible element of a manuscript.^9^ Recent studies have identified spin bias in SRMAs evaluating joint interventions such as platelet-rich therapy for hip osteoarthritis and stemless total shoulder arthroplasty.^10,11^ However, none have investigated spin bias in the context of RA-THA SRMAs.

The primary aim of this study was to evaluate the presence of spin bias in abstracts of SRMAs comparing RA-THA to C-THA and to examine study characteristics that are associated with increased spin bias.^6^ The secondary objective was to assess the methodological quality of the included studies using a validated quality assessment tool.^12^ We hypothesized that, consistent with prior orthopedic literature, spin bias would be prevalent and may exaggerate the perceived benefits of RA-THA, potentially influencing findings in favor of robotics.

## Methods

This systematic review was conducted in accordance with the Preferred Reporting Items for Systematic Reviews and Meta-Analyses guidelines (http://www.prisma-statement.org/). No institutional review board approval was required.

We conducted a comprehensive search of MEDLINE, EMBASE, and the Cochrane Database of Systematic Reviews databases from each databases’ inception to June 23, 2025. Search terms included “robotic,” “hip,” “arthroplasty,” “systematic review,” and “meta-analysis” (Supplemental Table 1).

The inclusion criteria were as follows: (1) SRMAs evaluating outcomes of RA-THA, (2) studies comparing RA-THA to conventional techniques, and (3) publications in English. SRMAs of all levels of evidence were included to achieve full comprehensiveness. Studies were excluded if they (1) assessed RA-THA with other robotic-assisted joint arthroplasty procedures, or (2) assessed RA-THA surgery alongside other technologies (eg navigation or computer assistance).

Two authors (A.M.A. and J.A.) screened the titles and abstracts of the retrieved studies in duplicate. Conflicts during title/abstract screening were advanced to the full-text review stage to avoid premature exclusion. Full-texts were then screened according to the aforementioned inclusion and exclusion criteria. Disagreements in full-text screening were resolved through consensus.

Two authors (A.M.A. and J.A.) independently extracted relevant study characteristics in duplicate using a premade template (Covidence, Veritas Health Innovation). Citation counts for each included SRMA were obtained from Web of Science on July 6, 2025. For each SRMA, all included primary studies were abstracted to identify the number of unique studies. Level of evidence was classified based on the guidelines by the *Journal of Bone and Joint Surgery.*^13^ Discrepancies during data abstraction were resolved in consultation with a senior author (H.A.K.).

To assess the presence of spin bias, the full text of each included SRMA was reviewed and compared with the results and conclusions presented in its abstracts. Each abstract was then assessed for spin bias using the 15 common domains described by Yavchitz et al,^6^ which are grouped into (1) misleading reporting, (2) misleading interpretation, and (3) inappropriate extrapolation. This assessment was performed independently and in duplicate by 2 reviewers (A.M.A. and J.A.) with prior training in assessment of spin bias. Conflicts were resolved through discussion with a senior author with graduate-level training in health research methodology (H.A.K.).

Quality assessment of the SRMAs included in our review was performed independently and in duplicate by 2 reviewers (A.M.A. and J.A.) using A Measurement Tool to Assess Systematic Reviews (AMSTAR-2).^12^ This tool is composed of 16 items, 7 of which are considered “critical” domains. Overall confidence in study quality is determined based on the number of critical and noncritical domains and is categorized as “high,” “moderate,” “low,” or “critically low.”^12^ Disagreements during the assessment were resolved in consultation with a senior author (H.A.K.).

### Statistical Analysis

Study summary characteristics were presented as descriptive statistics, including medians and interquartile range (IQR) or weighted means and standard deviations (SD), as appropriate. The frequency of unique studies as well as their level of evidence was also tabulated. Frequencies of spin bias domains and fulfilled AMSTAR-2 items were presented as counts and percentages. The frequencies of both the 9 (Spin-9) and 15 (Spin-15) most severe types of spin bias were tabulated to allow for comparisons against prior studies.^8,10^ Given that prior studies lacked adequate power for multivariable regression, study characteristics were compared with and without the most common types of spin bias using the Mann–Whitney U test for nonparametric data and the independent samples t-test for parametric data.^7,14^ Statistical analyses were performed using SPSS software, with significance set at *P* < .05.

## Results

**Figure 1.**
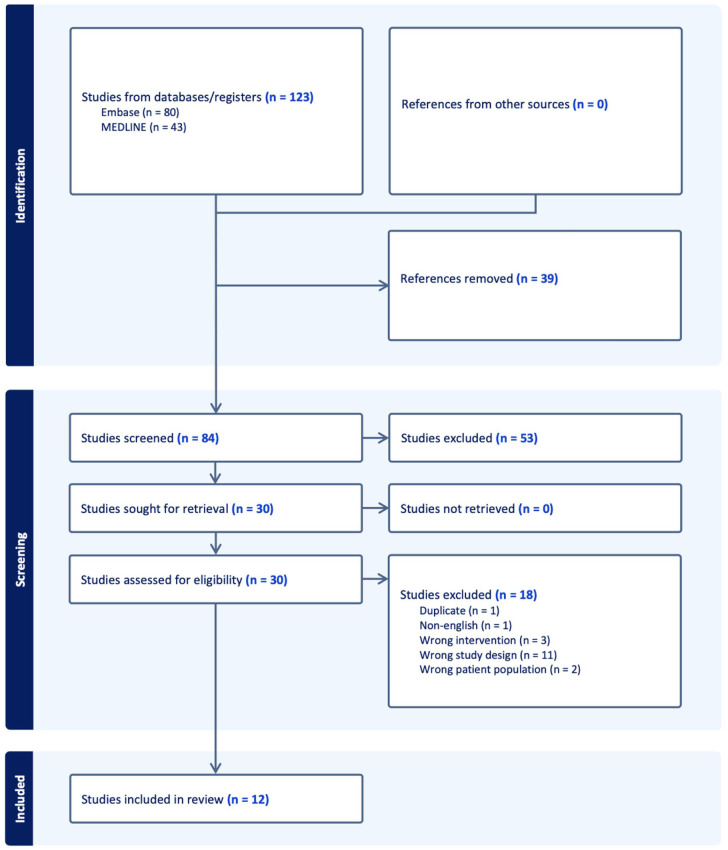
PRISMA. PRISMA, Preferred Reporting Items for Systematic Reviews and Meta-Analyses.

A total of 12 studies were included in the final analysis^15-26^ (Figure 1). The median sample size was 2828 hips (IQR, 1297-4243). The mean number of co-authors was 6.1 ± 1.6 (Table 1). The median number of citations per SRMA was 17.5 (IQR, 6.3 to 49.8) with a mean citation density of 8.8 (SD, 5.5). Across all SRMAs, 68 unique primary studies were included (Supplemental Table 2); 35 (51.5%) were cited in more than 1 SRMA. A randomized controlled trial (RCT) by Lim et al^27^ was the most frequently cited, appearing in 9 studies (75%); 6 other studies were included in 8 (66.7%) SRMAs.^28-33^

**Table 1.** Study Characteristics.

Author (Year)	Journal name (IF)	Total citations (Citation density)	Country	Authors, n	LOE	Number of studies, n (n hips)	Spin-9 present (Y/N)?	Spin-15 present (Y/N)?
Bensa et al. (2025)^25^	J Arthroplasty (3.8)	1 (1)	Switzerland	6	III	38 (10 214)	Yes	Yes
Chen et al. (2018)^24^	Postgrad Med J (2.7)	98 (14)	China	8	III	7 (1516)	Yes	Yes
Emara et al. (2021)^23^	Int J Med Robot (2.1)	50 (12.5)	United States	6	III	20 (4140)	Yes	Yes
Han et al. (2019)^22^	Int J Med Robot (2.1)	49 (8.2)	China	7	III	14 (2324)	Yes	Yes
Kumar et al. (2023)^21^	Postgrad Med J (2.7)	26 (13)	India	5	III	17 (3600)	Yes	Yes
Llombart-Blanco et al. (2024)^20^	J Orthop Surg Res (2.8)	6 (6)	Spain	5	IV	12 (1224)	Yes	Yes
Loke et al. (2025)^19^	J Orthop Surg Res (2.8)	0 (N/A)	Singapore	4	III	20 (6436)	Yes	Yes
Ng et al. (2021)^18^	Bone Joint J (4.6)	81 (20.3)	United Kingdom	6	III	17 (4278)	Yes	Yes
Ruangsomboon et al. (2024)^17^	J Robot Surg (3)	7 (7)	Canada	8	I	8 (977)	No	No
Samuel et al. (2022)^26^	J Robot Surg (3)	14 (3.5)	United States	5	III	18 (2811)	No	Yes
Sweet et al. (2021)^16^	JBJS Rev (2.4)	21 (5.3)	United States	4	III	7 (658)	Yes	Yes
Wang et al. (2023)^15^	Int J Med Robot (2.1)	13 (6.5)	China	9	III	18 (2845)	Yes	Yes

Abbreviations: LOE, Level of evidence; MA, Meta-analysis; SR, Systematic review; Spin-9 and Spin-15 represent the presence of ≥1 of the 9 and 15 most common spin domains described by Yavchitz et al, respectively.

The median journal impact factor was 2.8 (IQR, 2.2 to 3.0). The United States and China were the most common countries of origin, each publishing 3 studies. Most SRMAs were of Level III evidence (n = 10, 83.3%); only 1 systematic review did not also include a meta-analysis (Table 2). Nine (75%) of the SRMAs included at least 1 RCT^15-17,21-26^; collectively, there were 10 unique RCTs included across all eligible SRMAs.^27,31,32,34-40^ Most SRMAs were not funded (n = 7, 58.3%). Objective outcomes (n = 3, 25%) and resource utilization (n = 3, 25%) both being the least reported outcomes. Four studies (33.3%) specified robotic-specific eligibility criteria.^18-20,23^

**Table 2.** Frequencies of Study Characteristics.

Study characteristic	N (%)
Country	
Canada	1 (8.3)
China	3 (25)
India	1 (8.3)
Singapore	1 (8.3)
Spain	1 (8.3)
Switzerland	1 (8.3)
United Kingdom	1 (8.3)
United States	3 (25)
SR vs SR & MA	
SR	1 (8.3)
SR & MA	11 (91.7)
Funding	
Public	3 (25)
Private	2 (16.7)
Not funded	7 (58.3)
Robot-specific eligibility criteria	
Yes	3 (25)
No	9 (75)
LOE	
I	1 (8.3)
II	0 (0)
III	10 (83.3)
IV	1 (8.3)
Outcomes	
Patient reported outcomes	11 (91.7)
Objective outcomes (ie, range of motion)	3 (25)
Complications/reoperations	12 (100)
Intraoperative outcomes (ie, operative time, soft tissue injury)	11 (91.7)
Radiographic outcomes (includes implant positioning outcomes)	11 (91.7)
Resource utilization (ie, length of stay, cost)	3 (25)

Abbreviations: LOE, Level of evidence; MA, Meta-analysis; SR, Systematic review.

At least 1 of the 15 most severe forms of spin bias was present in the abstracts of 11 SRMAs (91.7%) (Table 1).^15,16,18-26^ The mean count of spin bias per study was 2 (SD, 1; range, 0-5). No differences in study characteristics were found across studies with 2 or less types of spin bias versus those with more than 2 (Supplemental Table 3). Ten studies (83.3%) presented with at least 1 form of the 9 most severe types of spin.^15,16,18-25^

Type 3 spin (selective reporting in favor of the intervention) was the most frequently occurring (n = 7, 58%), followed by type 5 (n = 6, 50%), and type 11 (n = 4, 33%) (Table 3). *Misleading reporting* and *misleading interpretation* spin categories were each present in 9 studies (n = 9, 75%). No study was found to have bias pertaining to *inappropriate extrapolation*. No significant differences in study characteristics were found between SRMA characteristics and the presence of the 3 most common types of spin bias (Type 3, 5, and 11; *P* > .05) (Supplemental Tables 4–6). While SRMAs with type 3 spin showed higher total citation counts (49.0 vs 13.0; *P* = .167), this difference became less pronounced when adjusting for publication year using citation density (9.6 vs 7.5; *P* = .575).

**Table 3.** Spin Classification.

Spin (category)	N (%)
1. Conclusion contains recommendations for clinical practice not supported by the findings (Misleading interpretation)	1 (8)
2. Title claims or suggests a beneficial effect of the experimental intervention not supported by the findings (Misleading interpretation)	0 (0)
3. Selective reporting of or overemphasis on efficacy outcomes or analysis favoring the beneficial effect of the experimental intervention (Misleading reporting)	7 (58)
4. Conclusion claims safety based on nonstatistically significant results with a wide confidence interval (Misleading interpretation)	2 (17)
5. Conclusion claims the beneficial effect of the experimental treatment despite high risk of bias in primary studies (Misleading interpretation)	6 (50)
6. Selective reporting of or overemphasis on harm outcomes or analysis favoring the safety of the experimental intervention (Misleading reporting)	0 (0)
7. Conclusion extrapolates the review’s findings to a different intervention (ie, claiming efficacy of 1 specific intervention although the review covers a class of several interventions) (Inappropriate extrapolation)	0 (0)
8. Conclusion extrapolates the review’s findings from a surrogate marker or a specific outcome to the global improvement of the disease (Inappropriate extrapolation)	0 (0)
9. Conclusion claims the beneficial effect of the experimental treatment despite reporting bias (Misleading interpretation)	3 (25)
10. Authors hide or do not present any conflict of interest (Misleading reporting)	2 (17)
11. Conclusion focuses selectively on statistically significant efficacy outcome (Misleading reporting)	4 (33)
12. Conclusion claims equivalence or comparable effectiveness for nonstatistically significant results with a wide confidence interval (Misleading interpretation)	2 (17)
13. Failure to specify the direction of the effect when it favors the control intervention	0 (0)
14. Failure to report a wide confidence interval of estimates	2 (17)
15. Conclusion extrapolates the review’s findings to a different population or setting (Inappropriate extrapolation)	0 (0)

All included SRMAs were rated as “critically low” confidence according to AMSTAR-2. Common methodological limitations included failure to provide a list of excluded studies with reasons (n = 12, 100%), lack of funding reporting within included studies (item 10; n = 11, 91.7%), and poor meta-analysis rigor (items 11, 12, and 13; n = 10, 90.9%) (Table 4). All studies reported inclusion criteria with Patient/Population, Intervention, Comparison, and Outcome components (item 1; n = 12, 100%), and most disclosed potential conflicts of interest (item 16; n = 10, 83.3%) (Supplemental Table 4).

**Table 4.** AMSTAR-2 Quality Assessment.

Domain	n (%)*		
	Yes	Partial yes	No
1. Did the research questions and inclusion criteria for the review include the components of PICO?	12 (100)	-	0 (0)
2. Did the report of the review contain an explicit statement that the review methods were established prior to the conduct of the review and did the report justify any significant deviations from the protocol?	7 (58.3)	0 (0)	5 (41.7)
3. Did the review authors explain their selection of the study designs for inclusion in the review?	4 (33.3)	-	8 (66.7)
4. Did the review authors use a comprehensive literature search strategy?	0 (0)	11 (91.7)	1 (8.3)
5. Did the review authors perform study selection in duplicate?	9 (75)	-	3 (25)
6. Did the review authors perform data extraction in duplicate?	7 (58.3)	-	5 (41.7)
7. Did the review authors provide a list of excluded studies and justify the exclusions?	0 (0)	0 (0)	12 (100)
8. Did the review authors describe the included studies in adequate detail?	2 (16.7)	4 (33.3)	6 (50)
9. Did the review authors use a satisfactory technique for assessing the RoB in individual studies that were included in the review?	6 (50)	2 (16.7)	4 (33.3)
10. Did the review authors report on the sources of funding for the studies included in the review?	1 (8.3)	-	11 (91.7)
^†^11. If meta-analysis was performed did the review authors use appropriate methods for statistical combination of results?	1 (9.1)	-	10 (90.9)
^†^12. If meta-analysis was performed, did the review authors assess the potential impact of RoB in individual studies on the results of the meta-analysis or other evidence synthesis?	1 (9.1)	-	10 (90.9)
13. Did the review authors account for RoB in individual studies when interpreting/ discussing the results of the review?	3 (25)	-	9 (75)
14. Did the review authors provide a satisfactory explanation for, and discussion of, any heterogeneity observed in the results of the review?	5 (41.7)	-	7 (58.3)
^†^15. If they performed quantitative synthesis did the review authors carry out an adequate investigation of publication bias (small study bias) and discuss its likely impact on the results of the review?	6 (54.5)	-	5 (45.5)
16. Did the review authors report any potential sources of conflict of interest, including any funding they received for conducting the review?	10 (83.3)	-	2 (16.7)

Abbreviations: PICO, Patient/Population, Intervention, Comparison, and Outcome; RoB, Risk of bias.

* “-” denotes response not applicable to domain.

† Denominator of studies adjusted to 11 as 1 study did not perform formal meta-analysis.

## Discussion

This study is the first to systematically evaluate the presence of spin bias in the abstracts of SRMAs on RA-THA. Importantly, 91.7% of included studies (n = 11) contained at least 1 of the 15 most severe spin types. Type 3 spin was most common (n = 7, 58%), followed by type 5 (n = 6, 50%) and type 11 (n = 4, 33%). On average, SRMAs presented with 2 counts of spin bias (range, 0-5). There were no differences in journal impact factor, publication year, citation counts or density, number of co-authors, number of hips included as well as number of studies included between SRMAs with <2 types of spin bias versus ≥2. Similarly, no differences in study characteristics were identified across SRMAs with and without the 3 most common types of spin bias. All included SRMAs were rated as “critically low” in methodological quality based on the AMSTAR-2 assessment.

This study has its limitations. First, the small number of eligible SRMAs combined with the high rates of spin bias precluded the performance of a multivariable logistic regression due to limited sample size and event rates. Nonetheless, we compared baseline study characteristics across SRMAs with and without the most common types of spin bias, in effort to identify possible drivers of this phenomenon. Second, AMSTAR-2 scoring is stringent and may disproportionately penalize older reviews that were less familiar with the tool, as key reporting standards (such as protocol registration and heterogeneity discussion) were less consistently applied.^22-24^ Finally, heterogeneity in eligibility criteria and inconsistent labeling of robotic systems rendered it impossible to compare semi-active with fully active systems, which may differ in outcome profiles but highlights the spin bias present in many of these studies. Nonetheless, this review highlighted important methodological considerations in RA-THA literature. Further, it assessed the 15 most severe forms of spin per Yavchitz et al, rather than the traditional 9.^6,8^ Ultimately, the results of this study highlight the importance of transparency in reporting as the adoption and evaluation of robotic technology in arthroplasty continues to grow.

The high prevalence of spin bias in our study is consistent with prior SRMAs on novel orthopedic interventions.^11,41^ For example, Biedermann et al^11^ reported spin bias in 83% of SRMAs on stemless shoulder arthroplasty, while Hwang et al^41^ found a rate of 87% in SRMAs on anterior cruciate ligament repair. These findings highlight that abstract-level bias can be common when evaluating orthopedic interventions. Type 3 spin, defined as “selective reporting of or overemphasis on efficacy outcomes or analysis favoring the beneficial effect of the experimental intervention,” was the most frequent form observed (56%). This aligns with prior orthopedic studies^7,8,42^ that have reported rates between 16% to 56%. Concerningly, our study’s prevalence of type 5 spin (50%)—claims of beneficial effects of the experimental treatment despite high risk of bias in the primary studies—was higher than the majority of prior studies,^7,8,14,42^ which ranged from 10% to 38%. The comparatively higher rate of type 5 spin in RA-THA SRMAs may reflect the limited evidence base, with most relying on a narrow pool of Level III studies sharing similar methodological limitations. Moreover, only 10 unique RCTs were collectively included across all eligible SRMAs,^27,31,32,34-40^ with only 3 published in the last 5 years.^37-39^ These findings underscore the need for future RA-THA SRMAs to present conclusions with greater caution and qualification, while also highlighting the need for stronger evidence through prospective RCTs.

Our analysis did not identify any study characteristics significantly associated with the presence of common spin bias types. However, a notable trend with types 3 and 11 spin bias was the selective emphasis on radiographic outcomes that favor RA-THA, particularly the proportion of acetabular components positioned within the Lewinnek safe zones. More specifically, our review often identified type 3 spin bias in the context of authors concluding that RA-THA improved radiological outcomes relative C-THA, citing significant results in parameters such as the Lewinnek safe zone, while omitting nonsignificant results in arguably more clinically relevant measures like cup inclination, stem positioning, and hip stability.^16,21,22,25^ This is of particular importance considering that the clinical relevance of Lewinnek safe zones has come into recent question.^43^ Given that most surgeons cite increased precision as the primary reason for adopting robotic-assisted technologies in arthroplasty,^44^ we hypothesize that the frequent overemphasis on component positioning may reflect an attempt to further align with this rationale for adoption. There may also be additional positive externalities to the adoption of robotic-assisted technologies in TJA, particularly in the physically demanding THA,^45^ that are not directly evaluated in studies to date, but may sway surgeon bias to favoring adoption of this technology. These include decreased surgeon strain^45^ as well as improved intraoperative ergonomics.^46^ Further, RA-THA may provide certain advantages in challenging patient populations, such as severely dysplastic hips, wherein the surgeon cannot rely on traditional intraoperative landmarks due to the patient’s abnormal anatomy.^47^ As such, spin bias identified by our review is limited to outcomes that have been amalgamated by SRMAs to date, which may not capture seldom reported surgeon-centric outcomes or outcomes in unique patient cohorts that may particularly benefit from this technology.

Another important caveat to consider is the heterogeneity in the robotic platforms that were evaluated across studies. Not all robotic systems function in the same way, yet this is often unclear in abstracts and conclusions. Only a quarter of included SRMAs assessed a predefined robotic system,^18-20^ specifically, the semi-active MAKO system that primarily assists with acetabular component positioning.^19^ Importantly, these studies avoided the third category of spin bias (*inappropriate extrapolation*) where authors might be tempted to generalize findings from MAKO to all robot systems. In contrast, first-generation, fully active systems such as ROBODOC target femoral stem preparation and positioning, and its results are reported across RA-THA literature.^31,34^ When SRMAs pool outcomes from both types of robots without clarifying these differences, particularly regarding stem positioning, conclusions about radiographic superiority can be misleading. Future RA-THA SRMAs should aim to minimize this heterogeneity and report system-specific analyses in their abstracts.

RA-THA SRMAs demonstrated critical methodological weaknesses, with all rated as “critically low” quality by AMSTAR-2 assessment. Unfortunately, critically low-quality SRMAs have commonly been identified across other orthopedic subspecialities.^7,8,14^ Of relevance is the lack of reporting on funding source, the second most common methodological limitation (n = 11, 91.6%). As demonstrated by Cavinatto et al,^48^ the rates of industry-funded studies were significantly higher in robotic-assisted unicompartmental knee arthroplasty studies compared to manual technique (51 vs 29%, *P* < .01). Concerningly, studies on robotic-assisted TJA with relevant author disclosures have been found to more likely present favorable results,^49^ with this trend corroborated across findings of health economic analyses of new technologies in TJA.^50^ Given these trends, it’s critical that future SRMAs on RA-THA present the funding sources of included studies as well as relevant author disclosures.

In conclusion, there is a high prevalence of spin bias in abstracts of SRMAs evaluating RA-THA. The most common type of spin bias involved selective reporting of outcomes favoring RA-THA (type 3). Future SRMAs can minimize spin bias by presenting balanced reporting of both efficacy or lack thereof in their abstracts and strengthen methodological rigor by consistently reporting study funding sources.

## Supplemental Material

sj-docx-2-hss-10.1177_15563316261430336 – Supplemental material for Evaluating Spin Bias in the Abstracts of Systematic Reviews and Meta-Analyses on Robotic-Assisted Total Hip Arthroplasty: A Systematic Review Supplemental material, sj-docx-2-hss-10.1177_15563316261430336 for Evaluating Spin Bias in the Abstracts of Systematic Reviews and Meta-Analyses on Robotic-Assisted Total Hip Arthroplasty: A Systematic Review by Ayomide Michael Ade-Conde, Hassaan Abdel Khalik, James Abesteh, Wayne E. Moschetti and Olufemi R. Ayeni in HSS Journal®

sj-docx-3-hss-10.1177_15563316261430336 – Supplemental material for Evaluating Spin Bias in the Abstracts of Systematic Reviews and Meta-Analyses on Robotic-Assisted Total Hip Arthroplasty: A Systematic Review Supplemental material, sj-docx-3-hss-10.1177_15563316261430336 for Evaluating Spin Bias in the Abstracts of Systematic Reviews and Meta-Analyses on Robotic-Assisted Total Hip Arthroplasty: A Systematic Review by Ayomide Michael Ade-Conde, Hassaan Abdel Khalik, James Abesteh, Wayne E. Moschetti and Olufemi R. Ayeni in HSS Journal®

sj-docx-4-hss-10.1177_15563316261430336 – Supplemental material for Evaluating Spin Bias in the Abstracts of Systematic Reviews and Meta-Analyses on Robotic-Assisted Total Hip Arthroplasty: A Systematic Review Supplemental material, sj-docx-4-hss-10.1177_15563316261430336 for Evaluating Spin Bias in the Abstracts of Systematic Reviews and Meta-Analyses on Robotic-Assisted Total Hip Arthroplasty: A Systematic Review by Ayomide Michael Ade-Conde, Hassaan Abdel Khalik, James Abesteh, Wayne E. Moschetti and Olufemi R. Ayeni in HSS Journal®

sj-docx-5-hss-10.1177_15563316261430336 – Supplemental material for Evaluating Spin Bias in the Abstracts of Systematic Reviews and Meta-Analyses on Robotic-Assisted Total Hip Arthroplasty: A Systematic Review Supplemental material, sj-docx-5-hss-10.1177_15563316261430336 for Evaluating Spin Bias in the Abstracts of Systematic Reviews and Meta-Analyses on Robotic-Assisted Total Hip Arthroplasty: A Systematic Review by Ayomide Michael Ade-Conde, Hassaan Abdel Khalik, James Abesteh, Wayne E. Moschetti and Olufemi R. Ayeni in HSS Journal®

sj-docx-6-hss-10.1177_15563316261430336 – Supplemental material for Evaluating Spin Bias in the Abstracts of Systematic Reviews and Meta-Analyses on Robotic-Assisted Total Hip Arthroplasty: A Systematic Review Supplemental material, sj-docx-6-hss-10.1177_15563316261430336 for Evaluating Spin Bias in the Abstracts of Systematic Reviews and Meta-Analyses on Robotic-Assisted Total Hip Arthroplasty: A Systematic Review by Ayomide Michael Ade-Conde, Hassaan Abdel Khalik, James Abesteh, Wayne E. Moschetti and Olufemi R. Ayeni in HSS Journal®

sj-pdf-1-hss-10.1177_15563316261430336 – Supplemental material for Evaluating Spin Bias in the Abstracts of Systematic Reviews and Meta-Analyses on Robotic-Assisted Total Hip Arthroplasty: A Systematic Review Supplemental material, sj-pdf-1-hss-10.1177_15563316261430336 for Evaluating Spin Bias in the Abstracts of Systematic Reviews and Meta-Analyses on Robotic-Assisted Total Hip Arthroplasty: A Systematic Review by Ayomide Michael Ade-Conde, Hassaan Abdel Khalik, James Abesteh, Wayne E. Moschetti and Olufemi R. Ayeni in HSS Journal®
