# Supplementary material for: Evaluating Spin Bias in the Abstracts of Systematic Reviews and Meta-Analyses on Robotic-Assisted Total Hip Arthroplasty: A Systematic Review
Source: HSS J. 2026 Apr 9:15563316261430336. Online ahead of print. doi: 10.1177/15563316261430336 (PMC13065638; doi:10.1177/15563316261430336)
Supplement: sj-docx-6-hss-10.1177_15563316261430336 – Supplemental material for Evaluating Spin Bias in the Abstracts of Systematic Reviews and Meta-Analyses on Robotic-Assisted Total Hip Arthroplasty: A Systematic Review [file sj-docx-6-hss-10.1177_15563316261430336.docx]

**Supplemental Digital Content Table 1** Search strategy

| MEDLINE, EMBASE and Cochrane Database of Systematic Reviews via OVID |
| --- |
| 1. exp Hip/  2. exp Hip Joint/  3. hip.ti,ab.  4. exp Arthroplasty/  5. exp Arthroplasty, Replacement/  6. exp Arthroplasty, Replacement, Hip/  7. arthroplasty.ti,ab.  8. replacement.ti,ab.  9. tha.ti,ab.  10. thr.ti,ab.  11.exp Joint Prosthesis/  12. prosthes*.ti,ab.  13. exp Robotic Surgical Procedures/  14. robot*.ti,ab.  15. robotic-assisted.ti,ab.  16. robotic arm-assisted.ti,ab.  17. robot-assisted.ti,ab.  18. robotic-arm.ti,ab.  19. robotic.ti,ab.  20. robot.ti,ab.  21. systematic review.ti,ab.  22. systematic-review.ti,ab.  23. meta analysis.ti,ab.  24. meta-analysis.ti,ab.  25. exp “Systematic Review”/  26. exp Meta-Analysis/  27. 1 or 2 or 3  28. 4 or 5 or 6 or 7 or 8 or 9 or 10 or 11 or 12  29. 13 or 14 or 15 or 16 or 17 or 18 or 19 or 20  30. 21 or 22 or 23 or 24 or 25 or 26  31. 27 and 28 and 29 and 30 |

**Supplemental Digital Content Table 2** Included studies across all systematic reviews and meta-analyses organized from most to least often included

| **Author et al (Year)** | **RCT vs non-RCT** | **Bensa et al (2025) [1]** | **Chen et al (2018) [2]** | **Emara et al (2021) [3]** | **Han et al (2019) [4]** | **Kumar et al (2023) [5]** | **Llombart-Blanco et al (2024) [6]** | **Loke et al (2025) [7]** | **Ng et al (2021) [8]** | **Ruangsomboon et al (2024) [9]** | **Samuel et al (2022) [10]** | **Sweet et al (2021) [11]** | **Wang et al (2023) [12]** | **Total, n (% of all)** |
| --- | --- | --- | --- | --- | --- | --- | --- | --- | --- | --- | --- | --- | --- | --- |
| Lim et al (2015) [13] | RCT | X | X | X | X | X |  |  |  | X | X | X | X | 9 (75) |
| Domb et al (2014) [14] | non-RCT | X | X | X | X | X | X |  | X |  |  |  | X | 8 (67) |
| Domb et al (2020) [15] | non-RCT | X |  | X |  | X |  | X | X |  | X | X | X | 8 (67) |
| El Bitar et al (2015) [16] | non-RCT | X |  | X | X | X | X | X | X |  |  |  | X | 8 (67) |
| Honl et al (2003) [17] | RCT | X |  | X | X | X |  |  |  | X | X | X | X | 8 (67) |
| Nakamura et al (2010) [18] | RCT | X | X | X | X | X |  |  |  | X | X |  | X | 8 (67) |
| Tsai et al (2016) [19] | non-RCT | X |  | X | X | X | X | X | X |  |  |  | X | 8 (67) |
| Bargar et al (2018) [20] | RCT | X |  |  | X | X |  |  |  | X | X | X | X | 7 (58) |
| Kamara et al (2017) [21] | non-RCT | X |  |  | X | X | X |  | X |  | X |  | X | 7 (58) |
| Nishihara et al (2006) [22] | RCT | X | X | X | X |  |  |  |  | X | X |  | X | 7 (58) |
| Bargar et al (1998) [23] | RCT |  |  | X | X | X |  |  |  | X | X |  | X | 6 (50) |
| Domb et al (2015) [24] | non-RCT |  | X | X | X | X |  |  | X |  |  |  | X | 6 (50) |
| Hananouchi et al (2007) [25] | non-RCT |  | X | X | X |  |  |  |  |  | X | X | X | 6 (50) |
| Nakamura et al (2018) [26] | RCT | X |  | X |  |  |  |  |  | X | X | X | X | 6 (50) |
| Kayani et al (2019) [27] | non-RCT | X |  |  |  | X |  | X | X |  |  |  | X | 5 (42) |
| Kong et al (2020) [28] | non-RCT | X |  |  |  | X |  |  | X |  | X |  | X | 5 (42) |
| Kong et al (2020) [29] | non-RCT | X |  | X |  |  |  |  | X |  | X |  | X | 5 (42) |
| Banchetti et al (2018) [30] | non-RCT |  |  |  |  |  |  | X | X |  |  | X |  | 3 (25) |
| Clement et al (2021) [31] | non-RCT |  |  | X |  |  |  | X |  |  | X |  |  | 3 (25) |
| Hadley et al (2020) [32] | non-RCT | X |  | X |  |  |  |  |  |  | X |  |  | 3 (25) |
| Kayani et al (2019) [33] | non-RCT |  |  | X |  |  |  | X | X |  |  |  |  | 3 (25) |
| Peng et al (2019) [34] | non-RCT |  |  | X |  |  |  | X |  |  | X |  |  | 3 (25) |
| Perets et al (2020) [35] | non-RCT |  |  | X |  |  |  | X |  |  | X |  |  | 3 (25) |
| Siebel et al (2005) [36] | RCT |  | X |  | X |  |  |  |  |  |  |  | X | 3 (25) |
| Ando et al (2021) [37] | non-RCT |  |  |  |  | X | X |  |  |  |  |  |  | 2 (17) |
| Bukowski et al (2016) [38] | non-RCT |  |  |  |  |  |  |  | X |  | X |  |  | 2 (17) |
| Foissey et al (2023) [39] | non-RCT | X |  |  |  |  |  | X |  |  |  |  |  | 2 (17) |
| Fontalis et al (2023) [40] | non-RCT | X |  |  |  |  |  | X |  |  |  |  |  | 2 (17) |
| Heng et al (2018) [41] | non-RCT |  |  |  |  |  |  | X | X |  |  |  |  | 2 (17) |
| Illgen et al (2017) [42] | non-RCT | X |  |  |  |  |  |  | X |  |  |  |  | 2 (17) |
| Incesoy et al (2023) [43] | non-RCT | X |  |  |  |  |  | X |  |  |  |  |  | 2 (17) |
| Singh et al (2021) [44] | non-RCT |  |  |  |  | X |  |  |  |  | X |  |  | 2 (17) |
| Suarez Ahedo et al (2017) [45] | non-RCT |  |  |  |  |  |  | X | X |  |  |  |  | 2 (17) |
| Wang et al (2023) [46] | RCT | X |  |  |  |  |  |  |  | X |  |  |  | 2 (17) |
| Xu et al (2020) [47] | non-RCT | X |  | X |  |  |  |  |  |  |  |  |  | 2 (17) |
| Alessia-Mazzola et al (2024) [48] | non-RCT |  |  |  |  |  |  | X |  |  |  |  |  | 1 (8) |
| Avram et al (2023) [49] | non-RCT | X |  |  |  |  |  |  |  |  |  |  |  | 1 (8) |
| Buchan et al (2023) [50] | non-RCT | X |  |  |  |  |  |  |  |  |  |  |  | 1 (8) |
| Caldora et al (2020) [51] | non-RCT | X |  |  |  |  |  |  |  |  |  |  |  | 1 (8) |
| Chai et al (2020) [52] | non-RCT |  |  |  |  | X |  |  |  |  |  |  |  | 1 (8) |
| Coulomb et al (2023) [53] | non-RCT |  |  |  |  |  |  | X |  |  |  |  |  | 1 (8) |
| Cui et al (2020) [54] | non-RCT |  |  |  |  |  | X |  |  |  |  |  |  | 1 (8) |
| Guo et al (2022) [55] | non-RCT |  |  |  |  |  | X |  |  |  |  |  |  | 1 (8) |
| Karlin et al (2024) [56] | non-RCT |  |  |  |  |  |  | X |  |  |  |  |  | 1 (8) |
| Kirchner et al (2021) [57] | non-RCT | X |  |  |  |  |  |  |  |  |  |  |  | 1 (8) |
| Kolodychuk et al (2021) [58] | non-RCT | X |  |  |  |  |  |  |  |  |  |  |  | 1 (8) |
| Kunze et al (2022) [59] | non-RCT |  |  |  |  |  | X |  |  |  |  |  |  | 1 (8) |
| LaValva et al (2024) [60] | non-RCT |  |  |  |  |  |  | X |  |  |  |  |  | 1 (8) |
| Lawson et al (2019) [61] | Non-RCT |  |  |  |  |  |  |  | X |  |  |  |  | 1 (8) |
| Lu et al (2023) [62] | RCT | X |  |  |  |  |  |  |  |  |  |  |  | 1 (8) |
| Lu et al (2024) [63] | RCT | X |  |  |  |  |  |  |  |  |  |  |  | 1 (8) |
| Ma et al (2023) [64] | non-RCT | X |  |  |  |  |  |  |  |  |  |  |  | 1 (8) |
| Ma et al (2023) [65] | non-RCT | X |  |  |  |  |  |  |  |  |  |  |  | 1 (8) |
| Nakumara et al (2009) [66] | non-RCT |  |  |  | X |  |  |  |  |  |  |  |  | 1 (8) |
| Ong et al (2024) [67] | non-RCT | X |  |  |  |  |  |  |  |  |  |  |  | 1 (8) |
| Redmond et al (2015) [68] | non-RCT |  |  |  |  |  |  |  | X |  |  |  |  | 1 (8) |
| Rogers et al (2024) [69] | non-RCT |  |  |  |  |  |  | X |  |  |  |  |  | 1 (8) |
| Sato et al (2023) [70] | non-RCT | X |  |  |  |  |  |  |  |  |  |  |  | 1 (8) |
| Shaw et al (2022) [71] | non-RCT | X |  |  |  |  |  |  |  |  |  |  |  | 1 (8) |
| Shibanuma et al (2021) [72] | non-RCT |  |  |  |  | X |  |  |  |  |  |  |  | 1 (8) |
| Singh et al (2022) [73] | non-RCT |  |  |  |  |  | X |  |  |  |  |  |  | 1 (8) |
| Stewart et al (2022) [74] | non-RCT | X |  |  |  |  |  |  |  |  |  |  |  | 1 (8) |
| Tian et al (2023) [75] | non-RCT | X |  |  |  |  |  |  |  |  |  |  |  | 1 (8) |
| Wulamu et al (2021) [76] | non-RCT |  |  |  |  |  | X |  |  |  |  |  |  | 1 (8) |
| Xu et al (2021) [77] | non-RCT |  |  |  |  |  | X |  |  |  |  |  |  | 1 (8) |
| Zhang et al (2021) [78] | non-RCT |  |  |  |  |  | X |  |  |  |  |  |  | 1 (8) |
| Zhang et al (2022) [79] | non-RCT | X |  |  |  |  |  |  |  |  |  |  |  | 1 (8) |
| Zhang et al (2023) [80] | non-RCT | X |  |  |  |  |  |  |  |  |  |  |  | 1 (8) |
| Zhou et al (2021) [81] | non-RCT | X |  |  |  |  |  |  |  |  |  |  |  | 1 (8) |

“X” denotes the inclusion of unique primary study in SRMA

**REFERENCES for SDC Table 2**

1. Bensa A, Pagliazzi G, Miele A, Schiavon G, Cuzzolin M, Filardo G. Robotic-Assisted Total Hip Arthroplasty Provides Greater Implant Placement Accuracy and Lower Complication Rates, but Not Superior Clinical Results Compared to the Conventional Manual Approach: A Systematic Review and Meta-Analysis. *J Arthroplasty*. 2025;40(7):1921-1931. doi:10.1016/j.arth.2024.12.014

2. Chen X, Xiong J, Wang P, et al. Robotic-assisted compared with conventional total hip arthroplasty: systematic review and meta-analysis. *Postgrad Med J*. 2018;94(1112):335-341. doi:10.1136/postgradmedj-2017-135352

3. Emara AK, Samuel LT, Acuña AJ, Kuo A, Khlopas A, Kamath AF. Robotic-arm assisted versus manual total hip arthroplasty: Systematic review and meta-analysis of radiographic accuracy. *Int J Med Robot*. 2021;17(6):e2332. doi:10.1002/rcs.2332

4. Han PF, Chen CL, Zhang ZL, et al. Robotics-assisted versus conventional manual approaches for total hip arthroplasty: A systematic review and meta-analysis of comparative studies. *Int J Med Robot*. 2019;15(3):e1990. doi:10.1002/rcs.1990

5. Kumar V, Patel S, Baburaj V, Rajnish RK, Aggarwal S. Does robotic-assisted surgery improve outcomes of total hip arthroplasty compared to manual technique? A systematic review and meta-analysis. *Postgrad Med J*. 2023;99(1171):375-383. doi:10.1136/postgradmedj-2021-141135

6. Llombart-Blanco R, Mariscal G, Barrios C, Vera P, Llombart-Ais R. MAKO robot-assisted total hip arthroplasty: a comprehensive meta-analysis of efficacy and safety outcomes. *J Orthop Surg Res*. 2024;19(1):698. doi:10.1186/s13018-024-05199-5

7. Loke RWK, Lim YH, Chan YK, Tan BWL. MAKO robotic-assisted compared to conventional total hip arthroplasty for hip osteoarthritis: a systematic review and meta-analysis. *J Orthop Surg Res*. 2025;20(1):466. doi:10.1186/s13018-025-05866-1

8. Ng N, Gaston P, Simpson PM, Macpherson GJ, Patton JT, Clement ND. Robotic arm-assisted versus manual total hip arthroplasty : a systematic review and meta-analysis. *Bone Joint J*. 2021;103-B(6):1009-1020. doi:10.1302/0301-620X.103B6.BJJ-2020-1856.R1

9. Ruangsomboon P, Ruangsomboon O, Osman K, et al. Clinical, functional, and radiological outcomes of robotic assisted versus conventional total hip arthroplasty: a systematic review and meta-analysis of randomized controlled trials. *J Robot Surg*. 2024;18(1):255. doi:10.1007/s11701-024-01949-z

10. Samuel LT, Acuña AJ, Mahmood B, Emara AK, Kamath AF. Comparing early and mid-term outcomes between robotic-arm assisted and manual total hip arthroplasty: a systematic review. *J Robot Surg*. 2022;16(4):735-748. doi:10.1007/s11701-021-01299-0

11. Sweet MC, Borrelli GJ, Manawar SS, Miladore N. Comparison of Outcomes After Robotic-Assisted or Conventional Total Hip Arthroplasty at a Minimum 2-Year Follow-up: A Systematic Review. *JBJS Rev*. 2021;9(6). doi:10.2106/JBJS.RVW.20.00144

12. Wang Y, Wang R, Gong S, et al. A comparison of radiological and clinical outcomes between robotic-assisted and conventional total hip arthroplasty: A meta-analysis. *Int J Med Robot*. 2023;19(1):e2463. doi:10.1002/rcs.2463

13. Lim SJ, Ko KR, Park CW, Moon YW, Park YS. Robot-assisted primary cementless total hip arthroplasty with a short femoral stem: a prospective randomized short-term outcome study. *Comput Aided Surg*. 2015;20(1):41-46. doi:10.3109/10929088.2015.1076044

14. Domb BG, El Bitar YF, Sadik AY, Stake CE, Botser IB. Comparison of robotic-assisted and conventional acetabular cup placement in THA: a matched-pair controlled study. *Clin Orthop Relat Res*. 2014;472(1):329-336. doi:10.1007/s11999-013-3253-7

15. Domb BG, Chen JW, Lall AC, Perets I, Maldonado DR. Minimum 5-Year Outcomes of Robotic-assisted Primary Total Hip Arthroplasty With a Nested Comparison Against Manual Primary Total Hip Arthroplasty: A Propensity Score-Matched Study. *J Am Acad Orthop Surg*. 2020;28(20):847-856. doi:10.5435/JAAOS-D-19-00328

16. El Bitar YF, Stone JC, Jackson TJ, Lindner D, Stake CE, Domb BG. Leg-Length Discrepancy After Total Hip Arthroplasty: Comparison of Robot-Assisted Posterior, Fluoroscopy-Guided Anterior, and Conventional Posterior Approaches. *Am J Orthop (Belle Mead NJ)*. 2015;44(6):265-269.

17. Honl M, Dierk O, Gauck C, et al. Comparison of robotic-assisted and manual implantation of a primary total hip replacement. A prospective study. *J Bone Joint Surg Am*. 2003;85(8):1470-1478. doi:10.2106/00004623-200308000-00007

18. Nakamura N, Sugano N, Nishii T, Kakimoto A, Miki H. A comparison between robotic-assisted and manual implantation of cementless total hip arthroplasty. *Clin Orthop Relat Res*. 2010;468(4):1072-1081. doi:10.1007/s11999-009-1158-2

19. Tsai TY, Dimitriou D, Li JS, Kwon YM. Does haptic robot-assisted total hip arthroplasty better restore native acetabular and femoral anatomy? *Int J Med Robot*. 2016;12(2):288-295. doi:10.1002/rcs.1663

20. Bargar WL, Parise CA, Hankins A, Marlen NA, Campanelli V, Netravali NA. Fourteen Year Follow-Up of Randomized Clinical Trials of Active Robotic-Assisted Total Hip Arthroplasty. *J Arthroplasty*. 2018;33(3):810-814. doi:10.1016/j.arth.2017.09.066

21. Kamara E, Robinson J, Bas MA, Rodriguez JA, Hepinstall MS. Adoption of Robotic vs Fluoroscopic Guidance in Total Hip Arthroplasty: Is Acetabular Positioning Improved in the Learning Curve? *J Arthroplasty*. 2017;32(1):125-130. doi:10.1016/j.arth.2016.06.039

22. Nishihara S, Sugano N, Nishii T, Miki H, Nakamura N, Yoshikawa H. Comparison between hand rasping and robotic milling for stem implantation in cementless total hip arthroplasty. *J Arthroplasty*. 2006;21(7):957-966. doi:10.1016/j.arth.2006.01.001

23. Bargar WL, Bauer A, Börner M. Primary and revision total hip replacement using the Robodoc system. *Clin Orthop Relat Res*. 1998;(354):82-91. doi:10.1097/00003086-199809000-00011

24. Domb BG, Redmond JM, Louis SS, et al. Accuracy of Component Positioning in 1980 Total Hip Arthroplasties: A Comparative Analysis by Surgical Technique and Mode of Guidance. *J Arthroplasty*. 2015;30(12):2208-2218. doi:10.1016/j.arth.2015.06.059

25. Hananouchi T, Sugano N, Nishii T, et al. Effect of robotic milling on periprosthetic bone remodeling. *J Orthop Res*. 2007;25(8):1062-1069. doi:10.1002/jor.20376

26. Nakamura N, Sugano N, Sakai T, Nakahara I. Does Robotic Milling For Stem Implantation in Cementless THA Result in Improved Outcomes Scores or Survivorship Compared with Hand Rasping? Results of a Randomized Trial at 10 Years. *Clin Orthop Relat Res*. 2018;476(11):2169-2173. doi:10.1097/CORR.0000000000000467

27. Kayani B, Konan S, Huq SS, Ibrahim MS, Ayuob A, Haddad FS. The learning curve of robotic-arm assisted acetabular cup positioning during total hip arthroplasty. *Hip Int*. 2021;31(3):311-319. doi:10.1177/1120700019889334

28. Kong X, Yang M, Jerabek S, Zhang G, Chen J, Chai W. A retrospective study comparing a single surgeon’s experience on manual versus robot-assisted total hip arthroplasty after the learning curve of the latter procedure - A cohort study. *Int J Surg*. 2020;77:174-180. doi:10.1016/j.ijsu.2020.03.067

29. Kong X, Yang M, Li X, et al. Impact of surgeon handedness in manual and robot-assisted total hip arthroplasty. *J Orthop Surg Res*. 2020;15(1):159. doi:10.1186/s13018-020-01671-0

30. Banchetti R, Dari S, Ricciarini ME, et al. Comparison of conventional versus robotic-assisted total hip arthroplasty using the Mako system: An Italian retrospective study. *J Health Soc Sci*. 2018;(1). doi:10.19204/2018/cmpr4

31. Clement ND, Gaston P, Bell A, et al. Robotic arm-assisted versus manual total hip arthroplasty. *Bone Joint Res*. 2021;10(1):22-30. doi:10.1302/2046-3758.101.BJR-2020-0161.R1

32. Hadley CJ, Grossman EL, Mont MA, Salem HS, Catani F, Marcovigi A. Robotic-Assisted versus Manually Implanted Total Hip Arthroplasty: A Clinical and Radiographic Comparison. *Surg Technol Int*. 2020;37:371-376.

33. Kayani B, Konan S, Thakrar RR, Huq SS, Haddad FS. Assuring the long-term total joint arthroplasty: a triad of variables. *Bone Joint J*. 2019;101-B(1_Supple_A):11-18. doi:10.1302/0301-620X.101B1.BJJ-2018-0377.R1

34. Peng Y, Arauz P, Desai P, Byers A, Klemt C, Kwon YM. In vivo kinematic analysis of patients with robotic-assisted total hip arthroplasty during gait at 1-year follow-up. *Int J Med Robot*. 2019;15(5):e2021. doi:10.1002/rcs.2021

35. Perets I, Walsh JP, Mu BH, et al. Short-term Clinical Outcomes of Robotic-Arm Assisted Total Hip Arthroplasty: A Pair-Matched Controlled Study. *Orthopedics*. 2021;44(2):e236-e242. doi:10.3928/01477447-20201119-10

36. Siebel T, Käfer W. [Clinical outcome following robotic assisted versus conventional total hip arthroplasty: a controlled and prospective study of seventy-one patients]. *Z Orthop Ihre Grenzgeb*. 2005;143(4):391-398. doi:10.1055/s-2005-836776

37. Ando W, Takao M, Hamada H, Uemura K, Sugano N. Comparison of the accuracy of the cup position and orientation in total hip arthroplasty for osteoarthritis secondary to developmental dysplasia of the hip between the Mako robotic arm-assisted system and computed tomography-based navigation. *Int Orthop*. 2021;45(7):1719-1725. doi:10.1007/s00264-021-05015-3

38. Bukowski BR, Anderson P, Khlopas A, Chughtai M, Mont MA, Illgen RL. Improved Functional Outcomes with Robotic Compared with Manual Total Hip Arthroplasty. *Surg Technol Int*. 2016;29:303-308.

39. Foissey C, Batailler C, Coulomb R, et al. Image-based robotic-assisted total hip arthroplasty through direct anterior approach allows a better orientation of the acetabular cup and a better restitution of the centre of rotation than a conventional procedure. *Int Orthop*. 2023;47(3):691-699. doi:10.1007/s00264-022-05624-6

40. Fontalis A, Kayani B, Haddad IC, Donovan C, Tahmassebi J, Haddad FS. Patient-Reported Outcome Measures in Conventional Total Hip Arthroplasty Versus Robotic-Arm Assisted Arthroplasty: A Prospective Cohort Study With Minimum 3 Years’ Follow-Up. *J Arthroplasty*. 2023;38(7 Suppl 2):S324-S329. doi:10.1016/j.arth.2023.04.045

41. Heng YY, Gunaratne R, Ironside C, Taheri A. Conventional vs Robotic Arm Assisted Total Hip Arthroplasty (THA) Surgical Time, Transfusion rates, Length of Stay, Complications and Learning Curve. *J Arthritis*. 2018;07(04). doi:10.4172/2167-7921.1000272

42. Illgen RL, Bukowski BR, Abiola R, et al. Robotic-Assisted Total Hip Arthroplasty: Outcomes at Minimum Two-Year Follow-Up. *Surg Technol Int*. 2017;30:365-372.

43. Incesoy MA, Yildiz F, Pulatkan MA, et al. CT-based, robotic-arm assisted total hip arthroplasty (Mako) through anterior approach provides improved cup placement accuracy but no difference in clinical outcomes when compared to conventional technique. *Technol Health Care*. 2024;32(5):3681-3691. doi:10.3233/THC-231111

44. Singh V, Realyvasquez J, Simcox T, Rozell JC, Schwarzkopf R, Davidovitch RI. Robotics Versus Navigation Versus Conventional Total Hip Arthroplasty: Does the Use of Technology Yield Superior Outcomes? *J Arthroplasty*. 2021;36(8):2801-2807. doi:10.1016/j.arth.2021.02.074

45. Suarez-Ahedo C, Gui C, Martin TJ, Chandrasekaran S, Lodhia P, Domb BG. Robotic-arm assisted total hip arthroplasty results in smaller acetabular cup size in relation to the femoral head size: a matched-pair controlled study. *Hip Int*. 2017;27(2):147-152. doi:10.5301/hipint.5000418

46. Wang W, Zhang Z, Wang G, et al. Prospective randomized controlled trial on the accuracy of prosthesis positioning in total hip arthroplasty assisted by a newly designed whole-process robotic arm. *Int Orthop*. 2023;47(2):413-419. doi:10.1007/s00264-022-05501-2

47. Xu S, Bernardo LIC, Andy KS, Pang HN. Robotic-Arm Assisted Direct Anterior Total Hip Arthroplasty; Improving Implant Accuracy. *Surg Technol Int*. 2020;38:347-352. doi:10.52198/21.STI.38.OS1368

48. Alessio-Mazzola M, Colombo P, Barducci N, et al. Direct anterior approach with conventional instruments versus robotic posterolateral approach in elective total hip replacement for primary osteoarthritis: a case-control study. *J Orthop Traumatol*. 2024;25(1):9. doi:10.1186/s10195-024-00753-7

49. Avram GM, Prill R, Gurau CD, et al. Acetabular cup placement and offset control in robotic total hip arthroplasty performed through the modified anterolateral approach. *Int Orthop*. 2023;47(9):2265-2273. doi:10.1007/s00264-023-05835-5

50. Buchan GBJ, Hecht CJ, Liu D, Mokete L, Kendoff D, Kamath AF. Improved accuracy of a novel fluoroscopy-based robotically assisted THA system compared to manual THA. *J Robot Surg*. 2023;17(5):2073-2079. doi:10.1007/s11701-023-01623-w

51. Caldora P, D’Urso A, Banchetti R, et al. Blood transfusion, hospital stay and learning curve in robotic assisted total hip arthroplasty. *J Biol Regul Homeost Agents*. 2020;34(4 Suppl. 3):37-49. Congress of the Italian Orthopaedic Research Society.

52. Chai W, Kong X, Yang M, Puah KL, Tang P, Chen J. Robot-Assisted Total Hip Arthroplasty for Arthrodesed Hips. *Ther Clin Risk Manag*. 2020;16:357-368. doi:10.2147/TCRM.S246565

53. Coulomb R, Cascales V, Haignere V, Bauzou F, Kouyoumdjian P. Does acetabular robotic-assisted total hip arthroplasty with femoral navigation improve clinical outcomes at 1-year post-operative? A case-matched propensity score study comparing 98 robotic-assisted versus 98 manual implantation hip arthroplasties. *Orthop Traumatol Surg Res*. 2023;109(1):103477. doi:10.1016/j.otsr.2022.103477

54. Cui K, Guo X, Chen Y, Zhong H, Han G, Liu Y. [A comparative study of MAKO robotic arm assisted total hip arthroplasty and traditional total hip arthroplasty through posterolateral approach]. *Zhongguo Xiu Fu Chong Jian Wai Ke Za Zhi*. 2020;34(7):883-888. doi:10.7507/1002-1892.201911077

55. Guo DH, Li XM, Ma SQ, Zhao YC, Qi C, Xue Y. Total Hip Arthroplasty with Robotic Arm Assistance for Precise Cup Positioning: A Case-Control Study. *Orthop Surg*. 2022;14(7):1498-1505. doi:10.1111/os.13334

56. Karlin E, Lee JW, Sanghavi K, Boucher H. Patient outcomes of conventional versus robot assisted total hip arthroplasty. *Current Orthopaedic Practice*. 2024;35(1):5-11. doi:10.1097/BCO.0000000000001244

57. Kirchner GJ, Lieber AM, Haislup B, Kerbel YE, Moretti VM. The Cost of Robot-assisted Total Hip Arthroplasty: Comparing Safety and Hospital Charges to Conventional Total Hip Arthroplasty. *J Am Acad Orthop Surg*. 2021;29(14):609-615. doi:10.5435/JAAOS-D-20-00715

58. Kolodychuk N, Su E, Alexiades MM, Ren R, Ojard C, Waddell BS. Can robotic technology mitigate the learning curve of total hip arthroplasty? *Bone Jt Open*. 2021;2(6):365-370. doi:10.1302/2633-1462.26.BJO-2021-0042.R1

59. Kunze KN, Huddleston HP, Romero J, Chiu YF, Jerabek SA, McLawhorn AS. Accuracy and Precision of Acetabular Component Position Does Not Differ Between the Anterior and Posterior Approaches to Total Hip Arthroplasty With Robotic Assistance: A Matched-Pair Analysis. *Arthroplast Today*. 2022;18:68-75. doi:10.1016/j.artd.2022.08.004

60. LaValva SM, Chiu YF, Fowler MJ, Lyman S, Carli AV. Robotics and Navigation Do Not Affect the Risk of Periprosthetic Joint Infection Following Primary Total Hip Arthroplasty: A Propensity Score-Matched Cohort Analysis. *J Bone Joint Surg Am*. 2024;106(7):582-589. doi:10.2106/JBJS.23.00289

61. Lawson JA, Garber AT, Stimac JD, Ramakrishnan R, Smith LS, Malkani AL. Does Robotic-Assisted Total Hip Arthroplasty Improve Accuracy of Cup Positioning? *J Hip Surg*. 2019;03(04):176-180. doi:10.1055/s-0039-1693480

62. Lu H, Sun H, Xiao Q, et al. Perioperative safety and efficacy of robot-assisted total hip arthroplasty in ERAS-managed patients: a pilot study. *J Orthop Surg Res*. 2023;18(1):696. doi:10.1186/s13018-023-04180-y

63. Lu X, Zhang Z, Xu H, Wang W, Zhang H. A new designed full process coverage robot-assisted total hip arthroplasty: a multicentre randomized clinical trial. *Int J Surg*. 2024;110(4):2141-2150. doi:10.1097/JS9.0000000000001103

64. Ma M, Cao Z, Yang M, Kong X, Chai W. The invasiveness of robot-assisted total hip replacement is similar to that of conventional surgery. *J Robot Surg*. 2023;17(6):2987-2993. doi:10.1007/s11701-023-01740-6

65. Ma M, Song P, Zhang S, Kong X, Chai W. Does robot-assisted surgery reduce leg length discrepancy in total hip replacement? Robot-assisted posterior approach versus direct anterior approach and manual posterior approach: a propensity score-matching study. *J Orthop Surg Res*. 2023;18(1):445. doi:10.1186/s13018-023-03864-9

66. Nakamura N, Sugano N, Nishii T, Miki H, Kakimoto A, Yamamura M. Robot-assisted primary cementless total hip arthroplasty using surface registration techniques: a short-term clinical report. *Int J Comput Assist Radiol Surg*. 2009;4(2):157-162. doi:10.1007/s11548-009-0286-1

67. Ong CB, Buchan GBJ, Hecht Ii CJ, Kendoff DO, Homma Y, Kamath AF. Fluoroscopy-based robotic assistance for total hip arthroplasty improves acetabular cup placement accuracy for obese patients compared to the manual, fluoroscopic- assisted technique. *Technol Health Care*. 2024;32(5):3703-3712. doi:10.3233/THC-231127

68. Redmond JM, Gupta A, Hammarstedt JE, Petrakos AE, Finch NA, Domb BG. The learning curve associated with robotic-assisted total hip arthroplasty. *J Arthroplasty*. 2015;30(1):50-54. doi:10.1016/j.arth.2014.08.003

69. Rogers N, Rullán PJ, Pasqualini I, et al. Lower 90-day inpatient readmission and 1-year reoperation in patients undergoing robotic versus manual total hip arthroplasty through an anterior approach. *Technol Health Care*. 2024;32(5):3769-3781. doi:10.3233/THC-231646

70. Sato K, Sato A, Okuda N, Masaaki M, Koga H. A propensity score-matched comparison between Mako robotic arm-assisted system and conventional technique in total hip arthroplasty for patients with osteoarthritis secondary to developmental dysplasia of the hip. *Arch Orthop Trauma Surg*. 2023;143(5):2755-2761. doi:10.1007/s00402-022-04524-z

71. Shaw JH, Rahman TM, Wesemann LD, Z Jiang C, G Lindsay-Rivera K, Davis JJ. Comparison of Postoperative Instability and Acetabular Cup Positioning in Robotic-Assisted Versus Traditional Total Hip Arthroplasty. *J Arthroplasty*. 2022;37(8S):S881-S889. doi:10.1016/j.arth.2022.02.002

72. Shibanuma N, Ishida K, Matsumoto T, et al. Early postoperative clinical recovery of robotic arm-assisted vs. image-based navigated Total hip Arthroplasty. *BMC Musculoskelet Disord*. 2021;22(1):314. doi:10.1186/s12891-021-04162-3

73. Singh A, Telagareddy K, Kumar P, Singh S. Robotic total hip arthroplasty for fused hips in ankylosing spondylitis patients: Our experience with robotic arm technology. *SICOT J*. 2022;8:30. doi:10.1051/sicotj/2022024

74. Stewart NJ, Stewart JL, Brisbin A. A Comparison of Component Positioning Between Fluoroscopy-Assisted and Robotic-Assisted Total Hip Arthroplasty. *J Arthroplasty*. 2022;37(8):1602-1605.e3. doi:10.1016/j.arth.2022.03.056

75. Tian R, Duan X, Kong N, Wang K, Yang P. Precise acetabular positioning, discrepancy in leg length, and hip offset using a new seven-axis robot-assisted total hip arthroplasty system requires no learning curve: a retrospective study. *J Orthop Surg Res*. 2023;18(1):236. doi:10.1186/s13018-023-03735-3

76. Wulamu W, Zhang X, Nuerailijiang Y, Ji B, Cao L. [Short-term effectiveness of Mako robot-assisted total hip arthroplasty via posterolateral approach]. *Zhongguo Xiu Fu Chong Jian Wai Ke Za Zhi*. 2021;35(10):1227-1232. doi:10.7507/1002-1892.202105120

77. Xu G, Ma M, Zhang S, Liu Y, Kong X, Chai W. [Application of Mako robot-assisted total hip arthroplasty in developmental dysplasia of the hip]. *Zhongguo Xiu Fu Chong Jian Wai Ke Za Zhi*. 2021;35(10):1233-1239. doi:10.7507/1002-1892.202105013

78. Zhang J, Wang X, Yang D, Zhou Y. [Early effectiveness of robot-assisted total hip arthroplasty via direct superior approach]. *Zhongguo Xiu Fu Chong Jian Wai Ke Za Zhi*. 2021;35(10):1240-1245. doi:10.7507/1002-1892.202105115

79. Zhang S, Liu Y, Yang M, et al. Robotic-assisted versus manual total hip arthroplasty in obese patients: a retrospective case-control study. *J Orthop Surg Res*. 2022;17(1):368. doi:10.1186/s13018-022-03263-6

80. Zhang S, Liu Y, Ma M, Cao Z, Kong X, Chai W. Is Robotic-Assisted Technology Still Accurate in Total Hip Arthroplasty for Fibrous-Fused Hips? *J Arthroplasty*. 2023;38(1):129-134. doi:10.1016/j.arth.2022.07.023

81. Zhou Y, Shao H, Huang Y, Deng W, Yang D, Bian T. Does robotic assisted technology improve the accuracy of acetabular component positioning in patients with DDH? *J Orthop Surg (Hong Kong)*. 2021;29(2):23094990211025325. doi:10.1177/23094990211025325

**Supplemental Digital Content Table 3** Comparison of study characteristics in articles with ≤ 2 versus >2 overall counts of spin

| **Study characteristic^†^** | **Spin** **≤ 2**  **(n=5)** | **Spin > 2**  **(n=7)** | **p-value** |
| --- | --- | --- | --- |
| Journal impact factor | 2.7 (2.1-3.3) | 2.8 (2.4-3.0) | 0.530 |
| Publication year^‡^ | 2021 ± 3 | 2023 ± 2 | 0.303 |
| Total citations | 49.5 (3.5-74.0) | 49.0 (6.0-50.0) | 0.570 |
| Citation density^‡^ | 8.3 ± 5.2 | 9.2 ± 6.3 | 0.802 |
| Number of co-authors^‡^ | 6.4 ± 1.1 | 5.9 ± 2.0 | 0.592 |
| Number of included studies | 14.0 (9.5-29.0) | 17.0 (8.0-18.0) | 0.935 |
| Number of included hips | 2324 (1370-7177) | 2845 (977-4278) | 0.935 |

^†^All variables as median (interquartile range) with Mann Whitney U test performed to compare medians unless otherwise noted

^‡^Presented as mean (standard deviation) with independent samples t-test performed to compare means

**Supplemental Digital Content Table 4** Comparison of study characteristics in articles with and without type 3 spin

| **Study characteristic^†^** | **No Spin (n=5)** | **Spin (n=7)** | **p-value** |
| --- | --- | --- | --- |
| Journal impact factor | 2.8 (2.4 – 3.0) | 2.7 (2.1 – 3.8) | .805 |
| Publication year^‡^ | 2023 ± 1 | 2021 ± 2 | .108 |
| Total citations | 13.0 (3.5 – 20) | 49.0 (81.0 – 6.0) | .167 |
| Citation density^‡^ | 7.5 ± 4.0 | 9.6 ± 6.4 | .575 |
| Number of co-authors^‡^ | 6.2 ± 2.2 | 6.0 ± 1.3 | .845 |
| Number of included studies | 18.0 (12.5 – 19.0) | 14.0 (7.0 – 20.0) | .462 |
| Number of included hips | 2845 (1894-5018) | 2324 (1224-4278) | .808 |

^†^All variables as median (interquartile range) with Mann Whitney U test performed to compare medians unless otherwise noted

^‡^Presented as mean (standard deviation) with independent samples t-test performed to compare means

**Supplemental Digital Content Table 5** Comparison of study characteristics in articles with and without type 5 spin

| **Study characteristic^†^** | **No Spin (n=6)** | **Spin (n=6)** | **p-value** |
| --- | --- | --- | --- |
| Journal impact factor | 2.9 (2.6 – 3.4) | 2.4 (2.1 – 3.1) | .145 |
| Publication year^‡^ | 2023 ± 2 | 2022 ± 3 | .452 |
| Total citations | 17.5 (5.3 – 39.8) | 31.0 (4.8 – 62.0) | .749 |
| Citation density^‡^ | 9.8 ± 6.8 | 8.0 ± 4.7 | .625 |
| Number of co-authors^‡^ | 5.3 ± 1.5 | 6.8 ± 1.5 | .112 |
| Number of included studies | 17.0 (7.8 – 18.5) | 16.0 (10.8 – 24.5) | .687 |
| Number of included hips | 3206 (897 – 4818) | 2585 (1443 – 5659) | .873 |

^†^All variables as median (interquartile range) with Mann Whitney U test performed to compare medians unless otherwise noted

^‡^Presented as mean (standard deviation) with independent samples t-test performed to compare means

**Supplemental Digital Content Table 6** Comparison of study characteristics in articles with and without type 11 spin

| **Study characteristic^†^** | **No Spin (n=8)** | **Spin (n=4)** | **p-value** |
| --- | --- | --- | --- |
| Journal impact factor | 2.8 (2.2 – 3.0) | 2.8 (2.2 – 3.0) | 1.00 |
| Publication year^‡^ | 2022 ± 2 | 2022 ± 3 | 0.793 |
| Total citations | 17.5 (8.5-43.3) | 28.0 (2.3-86.0) | .865 |
| Citation density^‡^ | 9.1 ± 5.7 | 8.4 ± 6.0 | .850 |
| Number of co-authors^‡^ | 6.0 ± 1.8 | 6.3 ± 1.3 | .815 |
| Number of included studies | 17.0 (9.5 – 18.0) | 16.0 (8.3 – 33.5) | .732 |
| Number of included hips | 2828 (1314-4109) | 2828 (1279-8696) | .734 |

^†^All variables as median (interquartile range) with Mann Whitney U test performed to compare medians unless otherwise noted

^‡^Presented as mean (standard deviation) with independent samples t-test performed to compare means

**Supplemental Digital Content Table 7** Study-specific AMSTAR-2 quality assessment

| **Author (year)** | **1** | **2** | **3** | **4** | **5** | **6** | **7** | **8** | **9** | **10** | **11** | **12** | **13** | **14** | **15** | **16** | **C** | **NC** | **Overall** |
| --- | --- | --- | --- | --- | --- | --- | --- | --- | --- | --- | --- | --- | --- | --- | --- | --- | --- | --- | --- |
| Bensa et al (2025) [1] |  |  |  |  |  |  |  |  |  |  |  |  |  |  |  |  | 4 | 4 | CL |
| Chen et al (2018) [2] |  |  |  |  |  |  |  |  |  |  |  |  |  |  |  |  | 3 | 5 | CL |
| Emara et al (2021) [10] |  |  |  |  |  |  |  |  |  |  |  |  |  |  |  |  | 5 | 5 | CL |
| Han et al (2019) [4] |  |  |  |  |  |  |  |  |  |  |  |  |  |  |  |  | 4 | 6 | CL |
| Kumar et al (2023) [5] |  |  |  |  |  |  |  |  |  |  |  |  |  |  |  |  | 5 | 4 | CL |
| Llombart-Blanco et al (2024) [6] |  |  |  |  |  |  |  |  |  |  |  |  |  |  |  |  | 2 | 5 | CL |
| Loke et al (2025) [7] |  |  |  |  |  |  |  |  |  |  |  |  |  |  |  |  | 3 | 5 | CL |
| Ng et al (2021) [8] |  |  |  |  |  |  |  |  |  |  |  |  |  |  |  |  | 4 | 4 | CL |
| Ruangsomboon et al (2024) [9] |  |  |  |  |  |  |  |  |  |  |  |  |  |  |  |  | 3 | 1 | CL |
| Samuel et al (2022) [10] |  |  |  |  |  |  |  |  |  |  |  |  |  |  |  |  | 6 | 5 | CL |
| Sweet et al (2021) [11] |  |  |  |  |  |  |  |  |  |  |  |  |  |  |  |  | 2 | 2 | CL |
| Wang et al (2023) [12] |  |  |  |  |  |  |  |  |  |  |  |  |  |  |  |  | 5 | 6 | CL |

CL, critically low; L, low; Red indicates “No”; Yellow indicates “Partial Yes”; Green indicates “Yes”; Grey indicated “Not applicable”
